# *Rhodococcus rhodochrous* ATCC12674 Becomes Alkane-Tolerant upon GroEL2 Overexpression and Survives in the *n*-Octane Phase in Two Phase Culture

**DOI:** 10.1264/jsme2.ME14114

**Published:** 2014-12-10

**Authors:** Hayato Takihara, Chiaki Matsuura, Jun Ogihara, Noriyuki Iwabuchi, Michio Sunairi

**Affiliations:** 1 Laboratory of Molecular Microbiology, Department of Applied Biological Science, College of Bioresource Sciences, Nihon University, 1866 Kameino, Fujisawa, Kanagawa 252–8510, Japan; 2 Laboratory of Enzyme Chemistry, Department of Chemistry and Life Science, College of Bioresource Sciences, Nihon University, 1866 Kameino, Fujisawa, Kanagawa 252–8510, Japan

**Keywords:** GroEL2, organic solvent tolerance, *Rhodococcus*, alkane, cell translocation, cell surface property

## Abstract

We recently reported that the overexpression of GroEL2 played an important role in increasing the alkane tolerance of *Rhodococcus erythropolis* PR4. In the present study, we examined the effects of the introduction of *groEL2* on the alkane tolerance of other *Rhodococcus* strains. The introduction of *groEL2* into *Rhodococcus* strains led to increased alkane tolerance. The translocation of *R. rhodochrous* ATCC12674 cells to and survival in the *n*-octane (C8) phase in two phase culture were significantly enhanced by the introduction of *groEL2* derived from strain PR4, suggesting that engineering cells to overexpress GroEL2 represents an effective strategy for enhancing organic solvent tolerance in *Rhodococcus*.

The rhodococci are a group of Gram-positive bacteria that have useful industrial and/or ecological applications due to their diverse range of metabolic activities. Some rhodococci can degrade organic compounds, including xenobiotics such as PCBs, whereas others are capable of degrading various aliphatic and aromatic hydrocarbons (1, 2, 8–10, 13), making these organisms ideal candidates for use in white biotechnology (3) and/or bioremediation.

The water/*n*-octanol partition coefficient of organic solvents (log *P*_o/w_) is used as a toxicity index (5). An organic solvent with a log *P*_o/w_ value in the range of 1–4 is generally considered to be highly toxic. Furthermore, the shorter the alkane carbon chain length, the more toxic the alkane (5). Since alkanes are major targets for degradation in bioremediation, the toxicity of these compounds to rhodococci must be overcome by improving their organic solvent tolerance if these organisms are to be useful for bioremediation purposes.

We have been studying organic solvent tolerance in *Rhodococcus*, and previously reported that extracellular polysaccharides derived from *Rhodococcus* played major roles in increasing their tolerance to organic solvents (6). We also recently demonstrated that the overexpression of GroEL2, which resulted from the introduction of a *groEL2*-encoding plasmid, contributed significantly to growth enhancements in *R. erythropolis* PR4 in the alkane phase (14). These findings suggested that engineering rhodococci to overexpress GroEL2 via the introduction of plasmids may represent a useful strategy for increasing the alkane tolerance of these organisms because GroEL2 is a molecular chaperone that has been shown to directly and/or indirectly contribute to overall cellular responses that increase the organic solvent tolerance of strain PR4 (14). In order to develop an efficient strategy for increasing alkane tolerance that would be widely applicable to the rhodococci, we herein examined the effects of the introduction of *groEL2* and its subsequent overproduction on the alkane tolerance of various rhodococci in an effort to construct strains capable of surviving and/or growing in the presence of alkanes with shorter carbon chains.

To understand the overall relationship between alkane carbon length, growth, and cell localization, the localization of 46 *Rhodococcus* strains in two phase cultures containing alkanes of different carbon-chain lengths was examined, and the results are summarized in Table S1. In this experiment, *n*-octane (C8), *n*-dodecane (C12), *n*-hexadecane (C16), or pristane (C19) were used as representative alkanes of varying carbon-chain lengths. The respective log *P*_o/w_ values of *n-*octane, *n*-dodecane, *n*-hexadecane, and pristane were 5.2, 6.1, 8.3, and 9.3. A visible increase in turbidity in the alkane phase and/or the presence of cells at the interface between the aqueous and alkane phases was observed in two phase cultures containing C12, C16, and C19, but not C8. In more than 85% of the strains examined, cells translocated to the alkane phase in the presence of C16 and C19. In two phase cultures with C12, approximately 60% of the strains translocated to the alkane phase. In contrast, no strains translocated to the alkane phase in the presence of C8 because all of the strains tested grew poorly in the presence of this alkane. These results revealed a close relationship between the log *P*_o/w_ value (which is based on alkane carbon-chain length) of an alkane, growth, and translocation frequency in *Rhodococcus*.

We subsequently examined the alkane carbon-chain length threshold at which a shift in cell localization occurred. Although the alkane chain-length threshold at which cells transitioned from translocation to adhesion or could not be detected varied according to the strain tested, strains were more likely to transition from translocation to adhesion or to become undetectable under all conditions tested. These results suggested that many *Rhodococcus* strains could grow in the alkane phase and were capable of translocation from one phase to another depending on the carbon-chain length of the alkane.

To determine whether the increase in alkane tolerance associated with introduction of the complete coding sequence (CDS) of *groEL2* was limited to strain PR4 only, we introduced the complete CDS of *groEL2* into other *Rhodococcus* strains (11). Plasmid pK4 (4), an *E. coli*-*Rhodococcus* shuttle vector, was used to introduce the CDS. The plasmid availability of various *Rhodococcus* strains was evaluated in preliminary experiments (Table S1), and five strains were selected as hosts in subsequent experiments.

The localization of 10 transformants was examined in two phase cultures with C8, C12, C16, or C19 using phase-contrast microscopy, and the results are summarized in Table S2. Enhanced primary alkane tolerance was observed under 5 of the 10 conditions tested (5 strains × 2 genes). Briefly, regardless of the gene origin, the transformants of two alkane-sensitive strains (*R. rhodochrous* R-1 and R-2) (6) were able to grow in the alkane phase in which the corresponding wild-type strains could not. Furthermore, the primary alkane tolerance of *R. rhodochrous* ATCC12674 (pK4-EL2-1) was enhanced. These results suggested that the effects of introducing the complete CDS of *groEL2* were not limited to PR4.

Surprisingly, the turbidity of *R. rhodochrous* ATCC12674 (pK4-EL2-1) was visible in the alkane phase in C8-containing two phase culture. Individual *R. rhodochrous* ATCC12674 (pK4-EL2-1) cells could be distinguished microscopically in the C8 phase (Fig. 1A). In contrast, the cells of *R. rhodochrous* ATCC12674 (pK4-EL2-AT) were not observed in the C8 phase (Fig. 1B). *R. rhodochrous* ATCC12674 (pK4) or *R. rhodochrous* ATCC12674 (pK4-*Δ*EL2-1) cells were also not observed in the C8 phase (data not shown). These results indicated that *R. rhodochrous* ATCC12674 (pK4-EL2-1) was capable of translocating to and surviving in the C8 phase.

To better define the relationship between cell translocation to and growth in the alkane phase, we examined the ability of *R. rhodochrous* ATCC12674 transformants to survive in C8-containing two phase culture. Cell suspensions (*ca.* 5 × 10^4^ CFU mL^−1^ each) were prepared and resuspended in IB medium (12), after which C8 was added to each sample to a final concentration of 5% (v/v). Samples were incubated at 28°C with shaking (110 rpm) for 215 h. Survival was assessed by determining CFUs on IB plates, and the percentage survival of each strain was calculated according to the following formula: percentage survival = number of CFU after 215 h of cultivation/number of CFU at the beginning of cultivation × 100. The survival rate of *R. rhodochrous* ATCC12674 (pK4-EL2-1) was significantly higher than that of the other transformants (Table 1), suggesting that the introduction of the complete CDS of *groEL2* derived from PR4 enhanced the survival of *R. rhodochrous* ATCC12674 in the presence of C8.

To examine the expression of GroEL2, shotgun proteomic analyses of the various strains were performed as described previously based on the PR4 genome (14), and the results are presented in Table 1. The cell density of each culture was adjusted to a final concentration of 1 × 10^7^ CFU mL^−1^ before total protein extraction for data normalization, and the protein extraction efficiency was evaluated for each sample. A total of 10 μg of protein from the membrane-containing supernatant was then subjected to SDS-PAGE followed by LC-MS/MS analysis, as described previously (14). Amino acid sequences determined by LC-MS/MS were searched against the *R. erythropolis* PR4 genome database, and the relative abundance of GroEL2 expressed by each transformant was determined.

The relative abundance of GroEL2 was significantly higher in *R. rhodochrous* ATCC12674 (pK4-EL2-1) than in the three other strains examined. In addition, the survival rate of *R. rhodochrous* ATCC12674 (pK4-EL2-1) in the presence of C8 was significantly higher than those of the three other strains examined. These results suggested that the overexpression of GroEL2 due to the introduction of the complete CDS of *groEL2* derived from PR4 enhanced the alkane tolerance of *R. rhodochrous* ATCC12674. In contrast, the relative abundance of GroEL2 and the survival rate in the presence of C8 were similar for *R. rhodochrous* ATCC12674 (pK4-EL2-AT) and *R. rhodochrous* ATCC12674 (pK4). Given that the amino acid sequence of GroEL2 was 99% identical between *R. rhodochrous* ATCC12674 and *R. erythropolis* PR4, *groEL2* derived from *R. rhodochrous* ATCC12674 may have contributed to the increased alkane tolerance observed. The introduction of pK4-EL2-AT to *R. erythropolis* PR4 altered cell localization (Table S2); therefore, *groEL2* derived from *R. rhodochrous* ATCC12674 may have contributed to the increased alkane tolerance. The reason why *groEL2* was not expressed in *R. rhodochrous* ATCC12674 following the introduction of pK4-EL2-AT currently remains unknown. Unfortunately, the host ranges of currently available gene expression systems are limited; therefore, further research aimed at developing a gene expression system applicable to *Rhodococcus* is needed.

We previously reported that the overexpression/upregulation of GroEL2 could indirectly contribute to increased cell surface lipophilicity via protein refolding in *R. erythropolis* PR4 (14). A kinetic lipophilicity assay using the microbial transfer to the ionic liquid (MTIL) method (7) was used to evaluate the effects of the overexpression of GroEL2 derived from *R. erythropolis* PR4 on the cell surface lipophilicity of *R. rhodochrous* ATCC12674 grown in the two phase culture with C8 added. The initial removal rate (R_0_) from the *n*-hexadecane phase for *R. rhodochrous* ATCC12674 (pK4-EL2-1) grown in the presence of C8 was 0.0 ± 0.1 min^−1^ (Table 1). This value was almost the same as that previously determined for other translocated cells (7, 14). Although data could not be compared between *R. rhodochrous* ATCC12674 (pK4-EL2-1) and the three other transformants due to the insufficient growth of the three other strains, the R_0_ value suggested that the cell surface of *R. rhodochrous* ATCC12674 (pK4-EL2-1) was sufficiently lipophilic; its degree of lipophilicity was sufficient to promote immediate translocation to the alkane phase and the subsequent maintenance of translocation to the alkane phase.

We herein examined the effects of the introduction of *groEL2* and subsequent overexpression of GroEL2 on the alkane tolerance of various *Rhodococcus* strains. The results obtained confirmed that the introduction of *groEL2* and subsequent overexpression of GroEL2 led to an increase in alkane tolerance in some rhodococci. By utilizing the overexpression of GroEL2 via the introduction of a plasmid encoding *groEL2* derived from PR4, we constructed a highly alkane-tolerant strain capable of translocating to and surviving in the presence of C8. It is generally believed that the shorter the carbon-chain length of an alkane, the more toxic it will be to bacteria in aqueous-alkane two phase culture (5). C8 is one of the most toxic liquid alkanes; none of the wild-type strains examined in the present study were able to grow in its presence. Our results showed that engineering bacteria to overexpress GroEL2 represents an effective strategy for increasing their tolerance to highly toxic organic solvents while maintaining their activity. By combining strategies for enhancing bacterial tolerance to organic solvents (such as that demonstrated in this study) with strategies for regulating the spatial arrangement between substrates and cells of interest, either in culture (data will appear elsewhere) or within their niche in the natural environment, more efficient methods could be developed for use in white biotechnology and/or bioremediation.

## Supplemental materials



## Figures and Tables

**Fig. 1:**
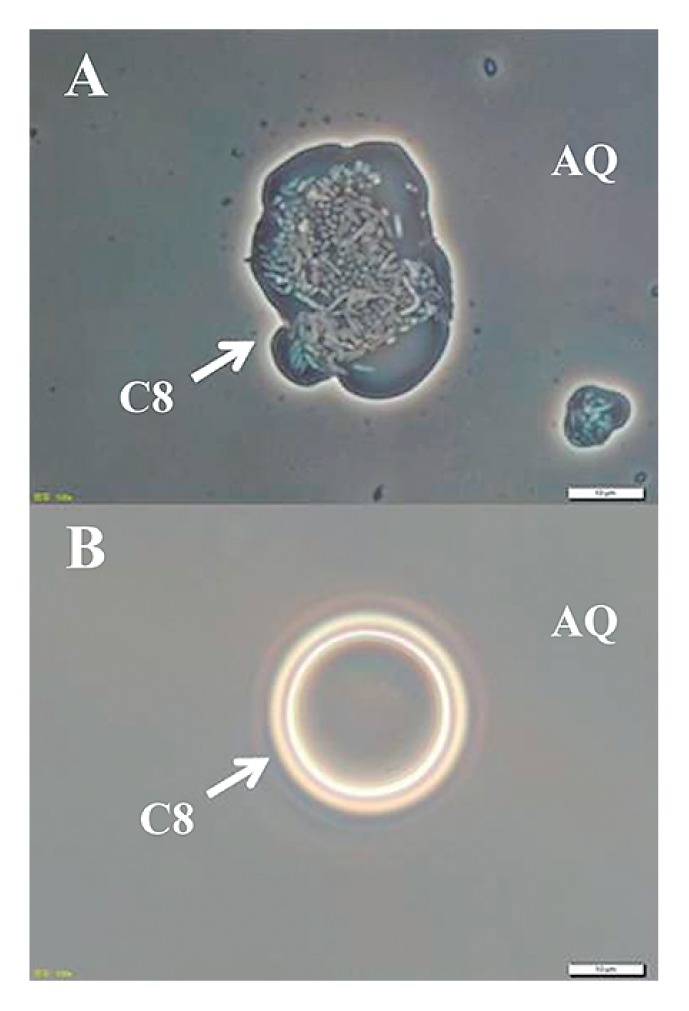
Phase-contrast micrographs illustrating the localization of *R. rhodochrous* ATCC12674 (pK4-EL2-1) and ATCC12674 (pK4-EL2-1-AT) grown in IB medium containing C8. A, ATCC12674 (pK4-EL2-1); B, ATCC12674 (pK4-EL2-1-AT). “C8” and “AQ” indicate the C8 phase and aqueous supernatant phase, respectively. Results shown are representative of 3 independent experiments, and 5 photographs were taken in each experiment (n=15). Both photographs were taken at the same magnification. Scale bar = 10 μm.

**Table 1:** Survival of *R. rhodochrous* ATCC12674 transformants, GroEL2 expression, and cell surface lipophilicity in two phase culture with C8

Plasmid	Survival rate (%)	GroEL2 (%)	R_0_ (min^−1^) measured by MTIL
pK4	8.8 (± 1.5)^*^	1.6 (± 1.8)^*^	N.D.
pK4-EL2-1	29.6 (± 3.3)	15.4 (± 0.1)	0.0 (± 0.1)
pK4-EL2-AT	8.9 (± 2.1)^*^	1.7 (± 1.0)^*^	N.D.
pK4-*Δ*EL2-1	11.0 (± 1.2)^*^	5.5 (± 0.7)^*^	N.D.

Plasmid descriptions: Plasmid pK4 is an *E. coli*-*Rhodococcus* shuttle vector (4), and plasmid pK4-EL2-1 contains the complete CDS of *groEL2* derived from PR4 at the *Eco*RI restriction site downstream of the kanamycin resistance gene, with the same respective forward orientation (14). Plasmid pK4-*Δ*EL2-1 was constructed by intramolecular self-ligation of a DNA fragment derived from *Aat*II-digested pK4-EL2-1, which did not code for the hinge region of GroEL2 (14). Plasmid pK4-EL2-AT contains the complete CDS of *groEL2* derived from strain ATCC12674.

Survival test: Values shown are the average and standard deviation (SD) of three independent experiments. Asterisks (*) represent significantly lower survival versus strain pK4-EL2-1; *P* < 0.05, Student’s *t*-test.

Shotgun proteomic analyses: Data represent the mean and SD determined from three independent experiments. Asterisks (*) represents significantly lower GroEL2 expression versus strain ATCC126744 (pK4-EL2-1); *P* < 0.05, Student’s *t*-test.

The kinetic lipophilicity assay was performed using the MTIL method as described previously (7). A tangent construction in the initial linear zone yields the initial removal rate R_0_ (min^−1^) as a measure of the adhesion of the bacteria to the ionic liquid, and the results are shown here. Data represent the mean and SD determined from three independent experiments. N.D. indicates that lipophilicity could not be determined because of insufficient growth of the tested strains.
