# Supplementary material for: Rhodococcus rhodochrous ATCC12674 Becomes Alkane-Tolerant upon GroEL2 Overexpression and Survives in the n-Octane Phase in Two Phase Culture
Source: Microbes Environ. 2014 Dec 10;29(4):431–3. doi: 10.1264/jsme2.ME14114 (PMC4262369; doi:10.1264/jsme2.ME14114)
Supplement: Supplementary file 1 [file 29_431_s1.pdf]

Supplemental materials

Rhodococcus rhodochrous ATCC12674 Becomes Alkane-Tolerant upon GroEL2 Overexpression and Survives in the *n*-Octane Phase in Two Phase Culture.

Hayato Takihara[1], Chiaki Matsuura[1], Jun Ogihara[2], Noriyuki Iwabuchi[1*], and Michio Sunairi[1].

[1]Laboratory of Molecular Microbiology, Department of Applied Biological Science, College of Bioresource Sciences, Nihon University, 1866 Kameino, Fujisawa, Kanagawa 252-8510, Japan; and [2]Laboratory of Enzyme Chemistry, Department of Chemistry and Life Science, College of Bioresource Sciences, Nihon University, 1866 Kameino, Fujisawa, Kanagawa 252-8510, Japan

Supplemental Tables

Table S1. Availability of plasmid pK4 and localization of *Rhodococcus* strains in two phase cultures containing alkanes of various carbon-chain lengths.

Genus and spices	Strain	Plasmid	C8	C12	C16	C19
Rhodococcus australis	ATCC35215	No	N.D.	Trn	Trn	Trn
Rhodococcus coprophilus	ATCC29080	No	N.D.	Trn	Trn	Trn
	JCM3200	No	N.D.	Trn	Trn	Trn
Rhodococcus erythropolis	ATCC27854	No	N.D.	Ahd	Trn	Trn
	ATCC47072	No	N.D.	Trn	Trn	Trn
	DSM1069	No	N.D.	Trn	Trn	Trn
	JCM3201	No	N.D.	Trn	Trn	Trn
	PR4	Yes	N.D.	Ahd*	Trn	Trn*
Rhodococcus globerulus	ATCC14346	No	N.D.	Ahd	Trn	Trn
	ATCC15076	No	N.D.	Trn	Trn	Trn
	ATCC21292	No	N.D.	Trn	Trn	Trn
	ATCC25669	No	N.D.	Trn	Trn	Trn
	ATCC25688	No	N.D.	Trn	Trn	Trn
	ATCC3110	No	N.D.	Trn	Trn	Trn
	ATCC14898	No	N.D.	Trn	Trn	Trn
Rhodococcus jostii	RHA1	Yes	N.D.	Trn	Trn	Trn
Rhodococcus opacus	ATCC170391	No	N.D.	Trn	Trn	Trn
	ATCC51881	No	N.D.	Ahd/Trn	Ahd	Trn
	ATCC51882	No	N.D.	Ahd	Trn	Trn
	JCM9703	No	N.D.	Trn	Trn	Trn

Rhodococcus percolatus	JCM10087	No	N.D.	Trn	Trn	Trn
Rhodococcus rhodnii	ATCC35071	No	N.D.	Trn	Trn	Trn
Rhodococcus rhodochrous	ATCC271	No	N.D.	Trn	Trn	Trn
	ATCC999	No	N.D.	Trn	Trn	Trn
	ATCC4001	No	N.D.	Ahd	Ahd	Trn
	ATCC13808	No	N.D.	Ahd	Ahd	Trn
	ATCC14348	No	N.D.	Ahd	Trn	Trn
	ATCC14349	No	N.D.	Trn	Trn	Trn
	ATCC15905	No	N.D.	Trn	Trn	Trn
	ATCC15906	No	N.D.	N.D.	Trn	Trn
	ATCC184	No	N.D.	Ahd	Trn	Trn
	ATCC17041	No	N.D.	Ahd	Trn	Trn
	ATCC19150	No	N.D.	Trn	Trn	Trn
	ATCC12674	Yes	N.D.	Trn	Trn	Trn
	JCM2156	No	N.D.	Trn	Trn	Trn
	JCM2157	No	N.D.	Trn	Trn	Trn
	R-1	Yes	N.D.	N.D.	Trn	Trn
	R-2	Yes	N.D.	N.D.	Trn	Trn
	S-1	Yes	N.D.	Ahd	Trn	Trn
	S-2	Yes	N.D.	Ahd	Ahd	Ahd
Rhodococcus ruber	IFO15591	No	N.D.	Ahd	Trn	Trn
Rhodococcus sp.	PG7-2	No	N.D.	Ahd	Trn	Trn
	JCM3376	No	N.D.	Trn	Trn	Trn
	JCM3391	No	N.D.	Ahd	Trn	Trn

Rhodococcus zopfii	ATCC51349	No	N.D.	Trn	Trn	Trn
	JCM9919	No	N.D.	Ahd	Trn	Trn

Four different alkanes were used in aqueous-alkane two phase cultures: *n*-octane (C8), *n*-dodecane (C12), *n*-hexadecane (C16), and pristane (C19), with log $P_{o/w}$ values of 5.2, 6.1, 8.3, and 9.3, respectively. The final alkane concentration in the two phase cultures was 5% (v/v). *Rhodococcus* strains were cultured in IB broth (12) at 28°C with shaking (110 rpm). Localization of cells was examined using phase-contrast microscopy (Olympus DP50, Tokyo, Japan). A total of 3 independent experiments were performed, and 5 photographs were taken in each experiment (n=15). Cell localization was determined based on which phase the majority of cells were located within in reference to Fig. S1. Ahd and Trn indicate adherence of cells at the interface between the aqueous-alkane phases and translocation of cells to the alkane phase, respectively. N.D. indicates that cell localization could not be determined because of insufficient growth of tested strains. *These data were compiled from a previous report (14).

Availability of the plasmid pK4 (4) in *Rhodococcus* strains was evaluated as follows: plasmid pK4 was introduced by electroporation as described previously (11). When required, strain-specific optimized conditions for electroporation were used. Plasmids were isolated from the transformants and examined by agarose gel electrophoresis. Yes and No indicate whether or not the introduced pK4 could be extracted from the transformant using standard methods.

Table S2. Localization of *Rhodococcus* transfromants in two phase cultures containing alkanes of various carbon-chain lengths.

Strain	Plasmid	C8	C12	C16	C19
PR4	None	N.D.	Ahd	Trn	Trn
	pK4-EL2-1	Ahd	Trn	Trn	Trn
	pK4-EL2-AT	Adh	Trn	Trn	N.T.
ATCC12674	None	N.D.	Trn	Trn	Trn
	pK4-EL2-1	Trn	Trn	Trn	Trn
	pK4-EL2-AT	N.D.	Trn	Trn	Trn
R-1	None	N.D.	N.D.	Trn	Trn
	pK4-EL2-1	N.D.	Ahd	Trn	Trn
	pK4-EL2-R1	N.D.	Ahd	Trn	Trn
R-2	None	N.D.	N.D.	Trn	Trn
	pK4-EL2-1	N.D.	Ahd	Trn	Trn
	pK4-EL2-R2	N.D.	Ahd	Trn	Trn
S-1	None	N.D.	Ahd	Trn	Trn
	pK4-EL2-1	N.D.	Ahd/Trn	Trn	Trn
	pK4-EL2-S1	N.D.	Ahd	Trn	Trn
S-2	None	N.D.	Ahd	Ahd	Ahd
	pK4-EL2-1	N.D.	Ahd	Ahd	Ahd
	pK4-EL2-S2	N.D.	Ahd	Ahd	Ahd

Plasmid construction and transformation were performed as described previously (11, 14). Plasmids pK4-EL2-AT, R1, R2, S1, and S2 encoded complete CDSs of *groEL2* derived from *R. rhodochrous* strains ATCC12674, R-1, R-2, S-1, and S-2, respectively.

Degenerate PCR primers (P1 = ATG GCI AAR ATH ATH GCI TTY GAY GAR GAR GCI MGI; P2 = RAA RTC CAT ICC ICC CAT ICC ICC IGT IGG RTC ICC) for amplification of *groEL2* CDSs of other *Rhodococcus* strains were constructed and used to determine the sequence of the cloned fragments. When required, a primer extension method was also used for determination of the sequence of the cloned fragments. The resulting DNA sequences were found to be identical to *groEL2*. The plasmid containing the cloned *groEL2* gene was digested using *Eco*RI, and the 1.6-kb *Eco*RI fragment was then inserted into the *Eco*RI restriction site of the *E. coli*-*Rhodococcus* shuttle vector pK4 (4).

Four different alkanes were used in aqueous-alkane two phase cultures: *n*-octane (C8), *n*-dodecane (C12), *n*-hexadecane (C16), and pristane (C19), with log $P_{o/w}$ values of 5.2, 6.1, 8.3, and 9.3, respectively. The final alkane concentration in the two phase cultures was 5% (v/v). *Rhodococcus* strains and their transformants were cultured in IB broth (12) at 28°C with shaking (110 rpm). When required, kanamycin was added to the IB medium at a concentration of 25-200 μg mL^{-1}.

Localization of cells in the two phase culture was examined using phase-contrast microscopy (Olympus DP50, Tokyo, Japan). A total of 3 independent experiments were performed, and 5 photographs were taken in each experiment (n=15). In reference to Fig. S1, cell localization was determined based on which phase the majority of cells were located within. Ahd and Trn indicate adherence of cells at the interface between the aqueous-alkane phases and translocation of cells into the alkane phase, respectively. N.D. indicates that cell localization could not be determined because of insufficient growth of the tested strains. N.T. indicates ‘not tested’.

Supplemental figure

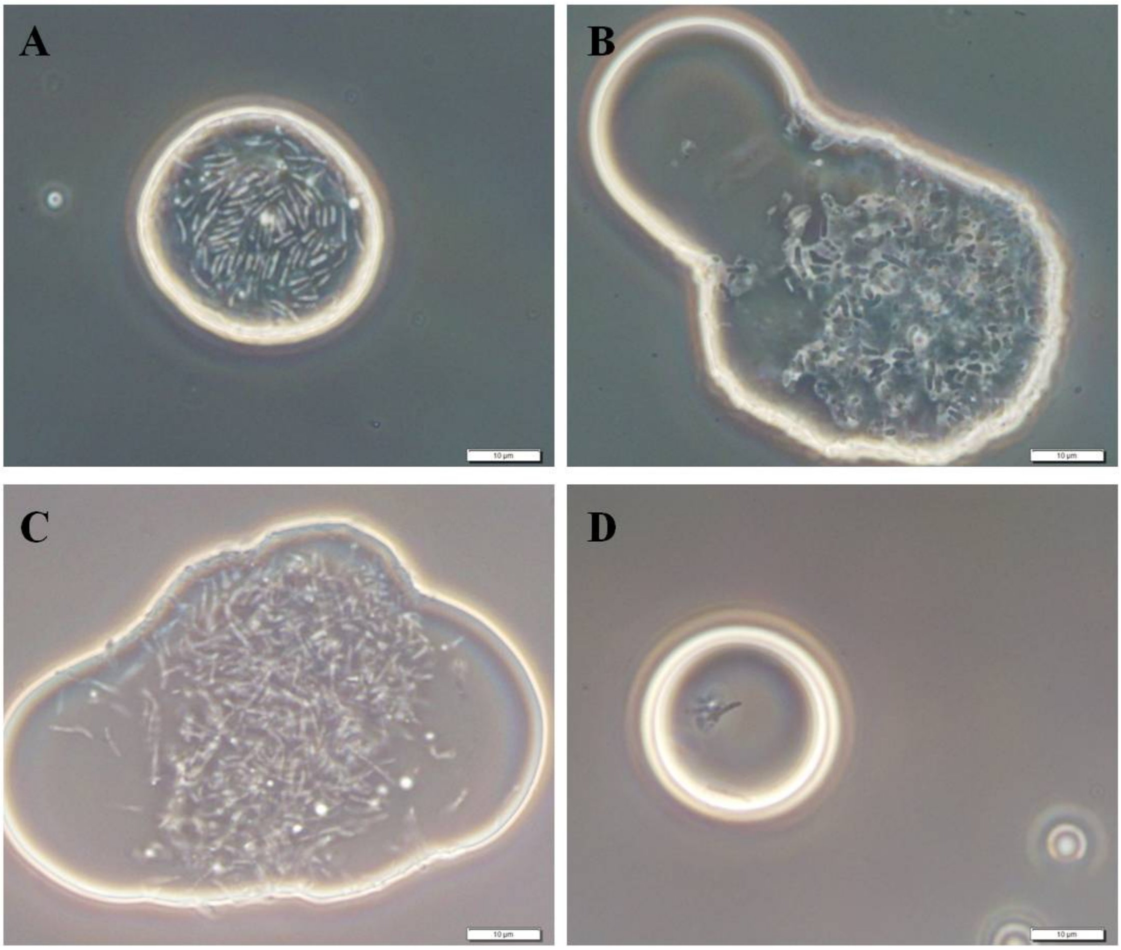

Fig. S1. Typical localization of *Rhodococcus* strains in alkane-containing two phase culture. Panels A, B, and C show representative photographs of translocated cells in the alkane phase, adherent cells at the aqueous-alkane interface, and both translocated and adherent cells, respectively. In panel D, no translocation of cells to the alkane phase was detected because the bacteria either grew poorly in the alkane phase and/or most of the cells remained in the aqueous phase. A, *R. erythropolis* PR4 grown in IB medium in the presence of C16; B, *R. rhodochrous* ATCC12674 grown in IB medium in the presence of C12; C, *R. opacus* ATCC51881 grown in IB medium in the presence of C12; D, *R. rhodochrous* S-2 grown in IB medium in the presence of C8.
